# The behavioural ecology of climbing plants

**DOI:** 10.1093/aobpla/plv013

**Published:** 2015-02-12

**Authors:** Ernesto Gianoli

**Affiliations:** 1 Departamento de Biología, Universidad de La Serena, Casilla 554, La Serena, Chile; 2 Departamento de Botánica, Universidad de Concepción, Casilla 160-C, Concepción, Chile

**Keywords:** Behavioural ecology, circumnutation, climbing plants, lianas, optimal foraging, support-searching, vines

## Abstract

Climbing plants require an external support to grow vertically and enhance light acquisition. Vines that find a suitable support have greater performance and fitness than those that remain prostrate. Therefore, the location of a suitable support is a key process in the life history of climbing plants. Numerous studies on climbing plant behaviour have elucidated mechanistic details of support searching and attachment. Far fewer studies have addressed the ecological significance of support-finding behaviour and the factors that affect it. Without this knowledge, little progress can be made in the understanding of the evolution of support-finding behaviour in climbers. Here I review studies addressing ecological causes and consequences of support finding and use by climbing plants. I also propose the use of behavioural ecology theoretical frameworks to study climbing plant behaviour. I show how host tree attributes may determine the probability of successful colonization for the different types of climbers, and examine the evidence of environmental and genetic control of circumnutation behaviour and phenotypic responses to support availability. Cases of oriented vine growth towards supports are highlighted. I discuss functional responses of vines to the interplay between herbivory and support availability under different abiotic environments, illustrating with one study case how results comply with a theoretical framework of behavioural ecology originally conceived for animals. I conclude stressing that climbing plants are suitable study subjects for the application of behavioural–ecological theory. Further research under this framework should aim at characterizing the different stages of the support-finding process in terms of their fit with the different climbing modes and environmental settings. In particular, cost–benefit analysis of climbing plant behaviour should be helpful to infer the selective pressures that have operated to shape current climber ecological communities.

## Abstract (precis)

Climbing plants require an external support to grow vertically and thus achieve better access to sunlight. Climbing plants that find a suitable support have greater performance, size and reproduction than those that remain prostrate. Plant behaviour involves rapid morphological or physiological responses to events or environmental changes. Theoretical frameworks from behavioural ecology, traditionally applied to animals, have been successfully used to study plant behaviour. I herein review studies addressing ecological causes and consequences of support finding and use by climbing plants. I also propose the use of behavioural ecology theoretical frameworks to study climbing plant behaviour.

## Introduction

Climbing plants need to attach themselves to an external support—typically neighbouring plants—in order to grow vertically to a significant extent and enhance light acquisition. Trellis availability influences climber diversity in forests (Garbin *et al*. 2012), and climbers that fail to encounter a trellis often show reduced growth and/or reproduction compared with those successfully climbing onto an external support. This has been observed in forests (Putz 1984; Stansbury *et al*. 2007), open habitats (Gianoli 2002; Price and Wilcut 2007; González-Teuber and Gianoli 2008) and controlled environments (Puntieri and Pyšek 1993; Schweitzer and Larson 1999). Support finding not only involves enhanced fitness but also triggers changes in growth form, biomass allocation, morphology and physiology (Raciborski 1900; Jaffe and Galston 1968*a*; Strong and Ray 1975; Ray 1987; Puntieri and Pyšek 1993; den Dubbelden and Oosterbeek 1995; Gianoli 2001, 2003). Therefore, the location (and colonization) of a suitable support is a key process in the life history of climbing plants (Hegarty 1991).

Darwin's observations on the oscillatory movements of exploring stems and tendrils (circumnutation) somehow founded the field of climbing plant behaviour (Darwin 1875). Since then, a plethora of studies on climbing plant behaviour with regard to support searching and attachment have elucidated mechanistic details at the anatomical, biomechanical, physiological and cellular levels (e.g. Tronchet 1945, 1946; Baillaud 1962; Jaffe and Galston 1968*a*; Millet *et al*. 1988; Putz and Holbrook 1991; Silk and Hubbard 1991; Brown 1993, Weiler *et al*. 1993; Scher *et al*. 2001; Kitazawa *et al*. 2005; Silk and Holbrook 2005; Goriely and Neukirch 2006; Bowling and Vaughn 2009; Stolarz 2009; Steinbrecher *et al*. 2010; Bauer *et al*. 2011; Gerbode *et al*. 2012). However, far fewer studies have addressed the ecological significance of support-finding behaviour in climbing plants and the factors that affect it (e.g. Peñalosa 1982; Larson 2000; Gianoli and Molina-Montenegro 2005; González-Teuber and Gianoli 2008). Without this knowledge, limited progress can be made in the understanding of the evolution of support-finding behaviour in climbers. Here, I review studies that have addressed ecological causes and consequences of support location and use by climbing plants. I also propose the use of behavioural ecology theoretical frameworks to study climbing plant behaviour. The article focusses mainly on twining plants, but also considers cases from plants having the other two ‘active’ modes of attachment: tendrils and adhesive roots (Darwin 1875; Isnard and Silk 2009).

## Ecological Approaches to Climbing Plant Behaviour

### Host tree characteristics

Several host tree attributes may determine the probability of colonization by climbers (Hegarty 1991). The size (diameter) of supports influences their suitability for twining plants. Specifically, both theoretical and empirical approaches show that when support diameter increases beyond some point twining plants are unable to maintain tensional forces and therefore lose attachment to the trellis (Putz 1984; Putz and Holbrook 1991; Goriely and Neukirch 2006; Carrasco-Urra and Gianoli 2009). That these plants have problems to twine round a thick support was already pointed out by Darwin, citing Hugo von Mohl's observations and reporting his own experiments with shoots of the twining vine *Wisteria sinensis* (Sims) Sweet, which could not climb onto a support nearly 15 cm wide (Darwin 1875). Field studies in tropical, subtropical and temperate rainforests confirm that the relative abundance of stem twiners decreases with increasing tree diameter (Putz 1984; Putz and Chai 1987; Carsten *et al*. 2002; Carrasco-Urra and Gianoli 2009). In a tropical rainforest, 90 % of stem twiners individuals with a diameter at breast height (dbh) of ≤1 cm grew on trees with a dbh of ≤8 cm (Nabe-Nielsen 2001). The support-size biomechanical constraints for twining plants are intermediate compared with tendril climbers, whose upper limit of usable trunk diameter is even lower, and root climbers, which are not constrained at all by large support diameters (Putz 1984; Putz and Chai 1987; Putz and Holbrook 1991; Chalmers and Turner 1994; DeWalt *et al*. 2000; Nabe-Nielsen 2001; Carrasco-Urra and Gianoli 2009). There is also a significant variation in the range of suitable support diameters within a given climbing mode. Thus, Peñalosa (1982) studied two twining lianas that differed in their degree of morphological specialization (shoot architecture) and found differential success rate of attachment across the population of support diameters in a tropical rainforest. As expected, vines may modify their climbing behaviour when twining around supports of different diameters. Thus, the ascent angle decreased with increasing support diameter in *Humulus lupulus* L. (Bell 1958) and *Dioscorea bulbifera* L. (Putz and Holbrook 1991; Scher *et al*. 2001), but the radius of curvature of the twining vine helix was unaffected. It has been suggested that the climber's coils lose stability when the radius of curvature of the helix is no longer greater than the support radius (Putz and Holbrook 1991).

Circumnutation behaviour and phenotypic responses to support availability, which may determine the suitable range of support sizes, should show—at least to some extent—environmental and genetic control. It has been shown that the shrub vs. vine growth forms in *Toxicodendron diversilobum* (Torr. & A.Gray) Greene are determined environmentally, mainly by support availability (Gartner 1991). Darwin (1875) noted that the twining vine *Phaseolus coccineus* L. failed to twine round sticks 8–10 cm in diameter when tested in a room with lateral light but the vines succeeded when placed outdoors. He further remarked that twiners from the tropics, or from warmer temperate regions, seemingly are able to ascend thicker trees (Darwin 1875). Whether twining plants from warmer habitats are better endowed to exploit thick supports is yet to be demonstrated, and should be addressed with a phylogeny-wise analysis since environment and species may be confounded factors. Interestingly, a recent study comparing climbing plants from temperate and subtropical South America found that a greater proportion of twiners occur in the subtropical, warmer area (Durigon *et al*. 2014). With regard to genetic variation, there is some evidence of differences in circumnutation behaviour and morphological plasticity in response to support availability between congeneric twining vines tested in a common environment (*Convolvulus* spp. and *Ipomoea* spp., Atala and Gianoli 2008; *Lonicera* spp., Schweitzer and Larson 1999). However, at the within-species level, the maternal family did not influence phenotypic responses to support availability in *I. purpurea* (L.) Roth (Gianoli and González-Teuber 2005). The quantitative trait loci controlling climbing ability have been identified in a recombinant inbred line of common bean, and most of these loci were found on the lower half of a given linkage group, suggesting the existence of a major pleiotropic locus controlling the climbing habit (Checa and Blair 2008). Experiments with mutants of *I. nil* (L.) Roth have demonstrated a link between circumnutation and gravisensing cells (Kitazawa *et al*. 2005).

Tree features such as bark roughness and flakiness may also influence support use by climbers (Putz 1980, 1984; Putz and Chai 1987; Campbell and Newbery 1993; Talley *et al*. 1996; Carsten *et al*. 2002; Campanello *et al*. 2007; van der Heijden *et al*. 2008). Bark flakiness has been considered an adaptation of trees against liana infestation, assuming that lianas may be unable to climb trees with rapidly shed bark because it implies loosing points of anchorage (Talley *et al*. 1996; Carsten *et al*. 2002). However, field evidence suggests that liana infestation is not particularly deterred in tree species that shed bark frequently (Carsten *et al*. 2002; Carrasco-Urra and Gianoli 2009; Jiménez-Castillo and Lusk 2009): climbers somehow manage to use as supports trees with peeling bark. On the other hand, the frequency of stem twiners in a rainforest did increase with bark roughness (Carsten *et al*. 2002). Interestingly, Darwin (1875) observed in kidney beans that the stem's axial twisting increased with support roughness, thus suggesting that twisted stems might be more rigid and that it could be advantageous to deal with rugged supports. Silk and Holbrook (2005) showed that the torsion of the twining stem was determined by helical parameters that vary with support diameter.

### Herbivory and support availability

Successful climbing by twining vines not only may help avoid shading by co-occurring taller plants, but also may place climbers beyond ground herbivores. There is field evidence that prostrate, unsupported vines suffer more herbivore damage than plants climbing onto neighbouring vegetation (Gianoli and Molina-Montenegro 2005; González-Teuber and Gianoli 2008; Gianoli and Carrasco-Urra 2014). Moreover, within a forest community, the identity of the supporting tree to which the climber is associated influences herbivore damage (Sasal and Suárez 2011; E. Gianoli and F. Carrasco-Urra, unpubl. data). In agreement with a hypothesis of adaptive climbing behaviour, it has been shown that circumnutation behaviour, measured as the twining rate on experimental supports, was enhanced in several Convolvulaceae vines receiving leaf damage (Gianoli and Molina-Montenegro 2005; Atala and Gianoli 2008). This induced twining—compared with undamaged plants—did not result from increased stem growth rate (Gianoli and Molina-Montenegro 2005; Atala and Gianoli 2008), which somewhat challenges the notion that circumnutation is intrinsically a growth movement (Mugnai *et al*. 2007). In *C. arvensis* L. the induced twining occurred similarly in both sun and shade conditions, and it was paralleled by an increase in photosynthetic rate, but only under shade (Gianoli and Molina-Montenegro 2005). This suggests that enhanced twining under low light entails an extra demand for resources by these vines. When stems of the twiner *C. chilensis* Pers. were clipped mimicking rabbit grazing in a semiarid shrubland, there was an increased production of tendril-like lateral stems that facilitated climbing in shade conditions (González-Teuber and Gianoli 2008). This phenomenon granted protection against herbivores by promoting the association with nurse plants (cacti and thorny shrubs); interestingly, such induction of tendril-like stems following damage only occurred in the shade, which is a cue of the presence of the nurse plant (González-Teuber and Gianoli 2008). Induced twining was also observed in *I. purpurea* after subjecting plants to folivory by snails as well as to exposure to conspecific volatiles (released from ground leaves) (Atala *et al*. 2014). In summary, the described phenomena of climbing plant behaviour in response to herbivory and abiotic conditions are likely to occur in natural ecological communities.

The exogenous application of jasmonic acid, a ubiquitous mediator of defensive responses in plants (Wasternack and Parthier 1997; Farmer *et al*. 2003), caused induced twining in *I. purpurea* as did leaf damage (Atala and Gianoli 2008). This could be a rather general response, as the application of jasmonate on the climbing plant *Bryonia dioica* caused tendril coiling (Falkenstein *et al*. 1991; Weiler *et al*. 1993). With regard to abiotic regulation of the phenomenon, water stress had contrasting effects on induced twining (Atala *et al*. 2011). On the one hand, moderate drought, which increases trichome density on stems of *I. purpurea* (Atala and Gianoli 2009*a*), enhanced the twining response (Atala *et al*. 2011). This result is consistent with the finding that trichomes facilitate climbing in this species as they function like ratchets (Silk and Holbrook 2005), which are analogous to hooks used by some climbing plants (Bauer *et al*. 2011). On the other hand, severe water stress limited the induced twining in *I. purpurea* (Atala and Gianoli 2009*b*; Atala *et al.* 2011), probably because extreme drought elicits plant responses that counteract phenotypic responses to herbivory (Quezada and Gianoli 2010).

Going back to the issue of the helical geometry of twining vines, Darwin (1875) noted that the terminal internodes made first a close spire, securing plant attachment during windy conditions, but the following spires were more open. This agrees with observations that a loosely coiled, old vine segment can be sustained by one or two tight younger coils (Putz and Holbrook 1991). It is also consistent with biomechanical experiments showing that forces pulling down a twining vine will tend to stabilize the plant–support interaction (i.e. the normal load exerted by the vine towards the support increases linearly with axial downward tension), unless the forces are applied close to the vine tip, because the twining vine is weak in compression (Silk and Holbrook 2005). Thus, grazing herbivores pulling down the climbing plant would not succeed: they would tear the vine before getting it to slip down. Interestingly, when documenting induced twining in *Ipomoea* vines, it was observed that leaf damage mimicking insect herbivory caused a reduction in the angle of ascent within the first three gyres in all tested species (*I. purpurea*, *I. tricolor* Cav., *I. nil*) (Atala and Gianoli 2008). Thus, vines respond to leaf damage as if they were twining around a thicker support (see above). Whether reduced angles of ascent in twiners translate into enhanced appression of the support remains to be tested, but related evidence suggests the opposite (Putz and Holbrook 1991; Silk and Hubbard 1991; Scher *et al*. 2001).

### Oriented growth and vine ‘decisions’

From an adaptive standpoint, an expected feature of climbing plant behaviour is that vines should be able to locate their supports and grow towards them. After conducting several simple experiments indoors, Darwin (1875) concluded that tendrils of *Bignonia capreolata* L. actively grow towards the dark, a phenomenon he later termed ‘apheliotropism’ (Darwin 1880). He remarked that circumnutation in these tendrils was extremely irregular, often staying static, that the apheliotropic movement was a modified circumnutation, and that this vine depended on apheliotropism to find tree trunks (Darwin 1880). One century later, experiments by Strong and Ray (1975) in a tropical forest showed that seedlings of the root climber *Monstera tenuis* K.Koch are attracted to the darkness, which is associated with the trees in the forest, and coined the term ‘skototropism’. Importantly, once the tree is found, the vine switches back and starts growing towards light (Strong and Ray 1975). Most documented cases of skototropism correspond to root climbers (Ray 1987; Hegarty 1991; Metcalfe 2005; Kato *et al*. 2012), but tendril-bearers can also exhibit this support-finding behaviour. Thus, apart from *B. capreolata* and its skototropic tendrils described by Darwin (1875), it has been reported that *Dolichandra unguis-cati* (L.) L.G.Lohmann (another Bignoniaceae) shows intra-plant variation in light response: the claw-like tendrils are skototropic and the shoot tips are positively phototropic (Lee and Richards 1991).

The above-described cases somewhat lend support to earlier claims that some climbing plants, including tendril-bearers (*Cyclanthera pedata* (L.) Schrad.) and stem twiners (*Cuscuta gronovii* Willd. ex Roem. & Schult.), were able to change their circumnutation patterns in order to reach support targets (Tronchet 1946, 1977); these reports were received with a degree of scepticism (Roussel 1978; Putz and Holbrook 1991). In the case of *Cuscuta* there is now solid evidence that these parasitic vines locate their host plants via oriented growth. Thus, experiments have proved that *Cuscuta* *pentagona* Engelm. seedlings grow towards regions of lowered red : far-red radiation ratio (a signal of the presence of chlorophyll-bearing organisms, Ballaré *et al*. 1987; Orr *et al*. 1996), and that they can locate host plants in the dark following volatile chemical cues (Runyon *et al*. 2006). The evolution of this highly specialized host location behaviour presumably results from strong selective pressures related to the parasitic lifestyle because, in order to survive, *Cuscuta* seedlings must attach to a host plant shortly after emergence; otherwise their energy reserves are exhausted. Thus, in a greenhouse experiment with autotrophic *Ipomoea* species there was no correlation between the red : far-red ratio in coloured stakes or corn plants and the frequency of vines twining around them (Price and Wilcut 2007).

Do community-level studies support the notion that climbers do not find support/hosts merely by chance? A number of field studies, conducted in almost all forest types, have shown associations between climbing plant species and tree species that are statistically different from what would be expected by chance (Hegarty 1991; Campbell and Newbery 1993; Chalmers and Turner 1994; Talley *et al*. 1996; Chittibabu and Parthasarathy 2001; Carsten *et al*. 2002; Muñoz *et al*. 2003; Nesheim and Økland 2007; Leicht-Young *et al*. 2010; Blick and Burns 2011). Consequently, host selection or host specificity has often been invoked to explain these patterns. An alternative explanation could consider the occurrence of convergence in microsite preference between vines and trees (Blick and Burns 2011). Apart from the tree traits possibly explaining differential susceptibility to vine infestation, the underlying mechanisms or the adaptive value of these patterns are rarely reported or discussed. This information is needed in order to determine the reliability and ecological significance of field patterns.

A central question that could be asked is whether vines actually make ‘decisions’ when it comes to support searching and selection. Apart from the evidence of oriented growth towards trees or experimental stakes discussed above, it could be added that climbing plants may reject a particular support. This was first described by Darwin for tendrils in *B. capreolata* initially seizing but then loosing sticks that were inappropriate (Darwin 1875). A similar phenomenon is observed when herbaceous twining vines get in contact with a very thick trunk and wind up on themselves instead of attempting to twine around it (a hopeless try, in view of the diameter constraints discussed above). In the case of annual vines, Darwin (1875) remarked that, even without support diameter constraints, it would be maladaptive to twine around thick—and hence large—trees, as these vines would hardly reach high-light layers by the end of the growing season.

The ‘self-twining’ (i.e. vine stems twining around each other) often occurs when vines grow beyond the height of a short support and then go up and down it, or when they fail to encounter a suitable support (Darwin 1875). What is the adaptive value of self-twining? To keep on circumnutating seems to be meaningless as new available supports would rarely appear, it would be energy-consuming, and—for a given species—circumnutation's range cannot be extended beyond some point because of biomechanical constraints: the diameters of circumnutation by shoot tips range from a few centimetres to over 1 m (Putz and Holbrook 1991). Ideally, twining vines should have a dual system, with trailing, skototropic stems searching for supports in addition to (or giving rise to) circumnutating stems. However, the closest known case did not prove efficient in this regard. Thus, the twining liana *I. phillomega* (Vell.) House produces creeping shoots (stolons) with high elongation rates under shade conditions, while twining shoots are produced in high-light conditions; but stolons did not switch to twining stems once support was found (Peñalosa 1983). A more efficient strategy is deployed by *Syngonium* root climbers, where a slender, skototropic prostrate stem searches for supports across the forest floor, but if a tree is not found after ∼2 m of extension (∼30 internodes), the plant reverts to the original, ‘sessile’ rosette form; the shoot alternates indefinitely between both forms until a tree is located (Ray 1987). Overall, climbers rarely show such functional division of labour among orthotropic (vertical) and plagiotropic (horizontal) shoots (Larson 2000; Gianoli 2001; Valladares *et al*. 2011). Some climbing plants seem to have a ‘give-up’ time concerning support finding. Darwin (1875) found that the twining hop (*H. lupulus*) stopped circumnutation after 5 days (37 revolutions) failing to find a support. Likewise, when the tip of a prostrate shoot of the climber *Lonicera sempervirens* touches the ground, circumnutation stops; it may resume after continued growth, but often in a different compass direction (Larson 2000). Long-lived species may have further chances: if searcher shoots of some lianas in a tropical rainforest fail to find a support, they fall over and are replaced by another shoot (Putz 1984).

## Theoretical Frameworks to Study Climbing Plant Behaviour

Plant behaviour involves rapid morphological or physiological responses to events or environmental changes (Karban 2008). Theoretical frameworks from behavioural ecology, traditionally applied to animals, have been successfully used to study plant behaviour (Marshall and Folsom 1991; Dudley and File 2007; Cahill and McNickle 2011; Jensen *et al*. 2011; Karst *et al*. 2012; Gagliano *et al*. 2014). Climbing plants have shown patterns of herbivory-induced chemical defences (Gianoli *et al*. 2007) that conform to optimal defence theory (Zangerl and Rutledge 1996). However, theoretical approaches from behavioural ecology have not been applied to the study of climbing plant behaviour. Ray (1992) described what he termed foraging behaviour in tropical Araceae climbers, characterizing shoot developmental patterns (length and diameter of internodes) in both trailing and climbing stems through the forest. In general terms, foraging behaviour in plants refers to their capacity of placing resource-acquiring structures (leaves and root tips) selectively within their habitat, where essential resources are usually heterogeneously distributed (Hutchings and De Kroon 1994). In the case of climbing plants, support finding brings about enhanced access to light resources (see above), but only then vines should fully display resource-acquiring structures. Accordingly, leaf expansion is delayed relative to stem extension in erect leader shoots of twiners and tendril climbers, thereby reducing stem load and facilitating support searching (Raciborski 1900; Baillaud 1962; French 1977). Field observations indicate that the maximum length these leafless leader shoots can attain before falling over (‘searcher shoot length’, Putz 1984) is species specific and largely determines the distance a climber can traverse between supports in the forest (Putz 1984). The capacity to span between supports is very important for the ecology of vines; however, to my knowledge, no study has specifically addressed phenotypic plasticity or evolutionary responses in searcher shoot length. In the following discussion of theoretical approaches to climbing plant behaviour, I will focus on foraging behaviour, specifically with regard to support searching.

**Figure 1.**
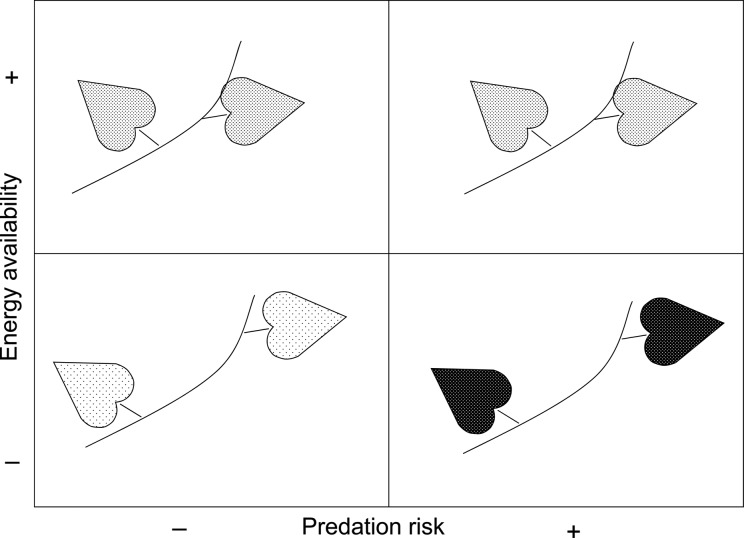
Anti-predator behaviour, measured as leaf alkaloid concentration (= density of points), and foraging behaviour, measured as internode length, in *C. arvensis* vines (Gianoli *et al*. 2007). Experimental plants were subjected to a factorial array of light availability (sun vs. shade) and support availability (climbing vs. prostrate vines). Field data indicate that predation (herbivory) risk is much higher on prostrate vines. Results verified the hypothesis that vines should show reduced anti-predator behaviour and increased foraging in the shade (fewer resources available). This agrees with animal-derived theory that posits that individuals are more willing to take predation risks during foraging when energetically stressed (Lima 1998).

Jensen *et al*. (2011) showed in the sensitive plant *Mimosa pudica* L., which rapidly folds its leaves when touched, that the anti-predator behaviour (time to leaf reopening after stimulation) was sustained longer under high-light conditions than under shading. This pattern supports predictions of animal-derived theory based on an optimality approach to anti-predation behaviour using stochastic dynamic programming (SDP) models (Lima 1998; Hutchinson and McNamara 2000). Specifically, the SDP model predicts that individuals are more willing to take predation risks during foraging when energetically stressed (Lima 1998). The theoretical framework of state-dependent decision-making under predation risk (Lima 1998) can be applied to a case of twining vines facing leaf damage with and without support availability and under contrasting abiotic environments. The study evaluated the effect of the light environment and support availability on the induction of tropane alkaloids (chemical defences) after leaf damage in *C. arvensis* (Gianoli *et al*. 2007), considering that herbivory pressure in the field is greater for prostrate vines compared with climbing vines (≈80 % vs. ≈40 % leaves showing damage; Gianoli and Molina-Montenegro 2005). The assumptions are that (i) alkaloid induction (difference between damaged and undamaged plants) is a measure of anti-predator behaviour, (ii) internode length is a proxy for vine foraging and (iii) the shade environment is where plants are energetically stressed. The prediction would be that vines should show reduced anti-predator behaviour and increased foraging in the shade (fewer resources available). However, this pattern should be observed only in the moderate herbivory scenario (climbing vines: 40 % leaf damage). In the case of prostrate vines growing under a strong herbivory pressure (80 % leaf damage), anti-predator behaviour should not be relaxed because it would be maladaptive, but enhanced vine foraging should hold. This is based on the fact that beyond some level of predation risk animals modify their (formerly adaptive) behaviour to adjust to the new environmental challenges (Lima and Dill 1990; Brown *et al*. 2006). Results of the twining vine study supported the predictions from the SDP model for optimal anti-predation behaviour under energy stress. Thus, in climbing vines (i.e. low predation risk) anti-predator behaviour was reduced and vine foraging was increased in the shade, while in prostrate vines (i.e. high predation risk) both anti-predator behaviour and vine foraging were greater in the shade (Gianoli *et al*. 2007) (Fig. 1). This analysis adds evidence to the notion that theoretical frameworks from animal behavioural ecology may also apply to plants.

Optimality models based on economic decisions have long been used to study animal foraging behaviour (Optimal Foraging theory; Emlen 1966; MacArthur and Pianka 1966; Charnov 1976; Pyke 1984; Krebs and Davies 1993) and may well be used to better understand climbing plant behaviour. In fact, Darwin, after concluding that—unlike tendrils—twining stems are not irritable, pointed out that it was not expected ‘as nature always economizes her means, and irritability would have been superfluous’ (Darwin 1875); this alludes to an optimality approach. The value of Darwin's analogies between plant strategies and economic concepts has been highlighted earlier (Drouin and Deroin 2010).

**Figure 2.**
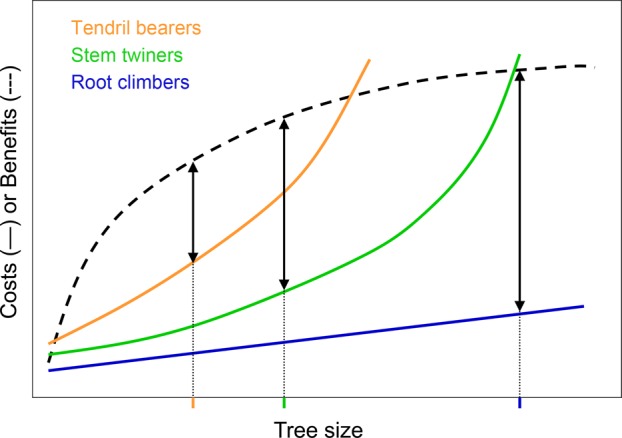
The hypothetical optimal tree size for vines (tree size includes both tree diameter and height) should vary with the climbing mechanism: tendril-bearers, stem twiners and root climbers. Benefits (∼ energy values) are assumed to increase with tree height because light harvest by climbing plants increases with height; the curve is further assumed to flatten out because most vines are not able to climb up to the top of canopy trees. Costs (∼ handling times) increase with tree diameter particularly for tendril-bearers and stem twiners because of biomechanical constraints: they fail to attach to thick trunks (see text); root climbers are free from this constraint but costs are assumed to increase slightly in very thick trunks—and hence old trees—because of the expected greater competition with other vines or epiphytes. Therefore, the optimal tree size, determined at the maximum distance between the cost and benefit curves, should be largest for root climbers and smallest for tendril-bearers.

The rationale behind the economics of prey choice for predators may be applied to climbing plants, with prey ≈ support, particularly focussing on the different modes of attachment (Putz and Chai 1987; Carsten *et al*. 2002; Llorens and Leishman 2008; Carrasco-Urra and Gianoli 2009). Actual prey value, which drives prey choice under an optimality approach, depends on the ratio between the prey's energy value (*E**_i_***) and the associated handling and search times (*h**_i_*** + *S**_i_***) (Krebs and Davies 1993). For vines, the support's energy value depends on light harvest after attaining maximum height on it, and hence could be roughly equated to tree height (Fig. 2). However, actual energy gain may be influenced by other extrinsic and intrinsic factors, such as canopy openness (determined by both the focus tree and the neighbouring trees) and intrinsic features of the vine (only a fraction of lianas reach the forest canopy, Putz 1980, 1984; Gerwing 2004). Handling time results in energy expenditure (Krebs and Davies 1993), and in the case of vines it is associated with the process of securing the attachment to the support. Handling time should increase with trellis diameter (≈ lower ascent angle, Bell 1958; Putz and Holbrook 1991; Scher *et al*. 2001) and vary with the degree of specialization of the climbing mechanism (Gentry 1991) (Fig. 2). For instance, both grasping by tendrils (Jaffe and Galston 1968*b*; Ma and Yen 1989) and circumnutation plus normal loads by stem twiners (Silk and Hubbard 1991; Stolarz 2009) are more ATP-consuming than the rather passive mechanisms of leaning on hosts shown by scrambling or hook climbers (Hegarty 1991; Isnard and Silk 2009). Searching time will depend on the density of trellises in a given habitat, taking into account that for some climbing mechanisms (tendril-bearers, stem twiners) thick supports are not suitable.

**Figure 3.**
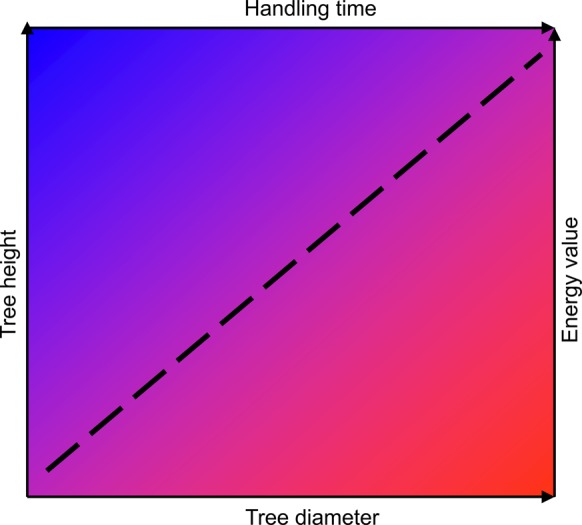
Components of support value (∼ prey profitability) for climbing plants may show a trade-off because of the intrinsic association between tree diameter and tree height (dashed line). Thin trees are easy to climb (low handling time) but result in short heights (low energy value), while the opposite occurs for thick trees. Highest and lowest support values are shown in blue and red, respectively.

Interestingly, components of support value may trade-off. Thus, thin supports may be easy to climb but result in a short final height for the vine (low handling time, low energy value), while thick supports may be hard to climb but result in a tall final height (high handling time, high energy value) (Fig. 3). Exceptions to this could be observed in those cases where vines ascend by climbing older vines (i.e. relatively thin and tall supports) (see Putz 1984). Trade-offs among components of foraging, particularly among those with a known—or assumed—relationship with fitness (fitness currency, Pyke 1984), are major constraints for the evolution of adaptive foraging behaviour (Krebs and Davies 1993). A more complex scenario may arise considering that fitness currencies may vary with the climbing mechanism and/or life history of vines. Thus, the premise that the energy value of the support depends on light harvest after attaining maximum height on it assumes that the plant aims at maximizing growth and carbon gain. However, field studies have shown that some vine species prioritize growth rate and carbon gain, while other species display traits enhancing survival in low light (Gilbert *et al*. 2006; Valladares *et al*. 2011; Gianoli *et al*. 2012). Moreover, the species' climbing mechanism influences its photosynthetic acclimation and abundance in contrasting light environments (Carter and Teramura 1988; Teramura *et al*. 1991) such as those found along the vertical light gradient in the forest.

All support's components taken together (energy value, handling time and search time) could lead to predictions of ‘favourite’ ecosystems for particular vine forms or species, and forest types and/or successional stages appear as good predictors of such differential suitability. There are some general patterns described in the literature, such as Durigon *et al*. (2014), but functional explanations are wanting. For instance, root climbers are not constrained by thick tree trunks (Putz and Holbrook 1991; Carrasco-Urra and Gianoli 2009), often show an efficient searching strategy for shaded habitats: skototropism (Hegarty 1991; Lee and Richards 1991), but their attachment to trees should be challenged in open and dry habitats because adventitious roots may suffer desiccation at high irradiances (Carter and Teramura 1988; Teramura *et al*. 1991). Therefore, it was no surprise to find that, globally, root climbers were more frequent in forests with greater precipitation and reduced seasonality, and that increasing temperature reduced root-climber occurrence in tropical sites (Durigon *et al*. 2013).

## Concluding Remarks

Climbing plants account for a significant component of plant evolution, diversity and abundance and play a major role in forest communities and ecosystems (Putz and Mooney 1991; Schnitzer and Bongers 2002; Gianoli 2004; Durán and Gianoli 2013; Schnitzer *et al*. 2015). Moreover, the relative abundance of woody climbers is increasing in tropical forests (Phillips *et al*. 2002; Schnitzer and Bongers 2011) and several of the most aggressive invasive plants worldwide are vines (Holm *et al*. 1991). Therefore, from several different standpoints it is of paramount importance to understand the ecological factors and physiological mechanisms that determine the vines' successful use of neighbouring vegetation as support.

In this overview I have identified main issues of climbing plant behaviour, most of them tracing back to Darwin's seminal observations, which deserve further ecological inquiry. Other aspects of climbing plant behaviour, such as patterns of twining handedness (Darwin 1875; Edwards *et al*. 2007; Burnham and Revilla-Minaya 2011) or support-finding benefits in desert vines, which grow in environments where light availability is not limiting (Rundel and Franklin 1991; Krings 2000; Parsons 2006), still wait for ecological explanations.

I have shown that climbing plants are suitable study subjects for the application of behavioural–ecological theory. Optimality models are particularly useful because they often provide testable, quantitative predictions (Krebs and Davies 1993). Further research under this theoretical framework should aim at characterizing the different stages of the support-finding process (search time, handling time) in terms of (i) their fit with the different climbing modes and environmental settings and (ii) their association with plant fitness. In particular, cost–benefit analysis of climbing plant behaviour should be helpful to infer the selective pressures that have operated to shape current climber ecological communities (see Rowe *et al*. 2004). This should be followed by phenotypic selection analyses of field data and the determination of the genetic basis of the key plant traits (e.g. Saldaña *et al*. 2007; Gianoli and Saldaña 2013) in order to understand their potential for evolutionary responses.

## Sources of Funding

The study was supported by FONDECYT (Fondo Nacional de Desarrollo Científico y Tecnológico—Chile) grant 1140070.

## Conflicts of Interest Statement

None declared.
